# Wafer-scale single-crystal perovskite patterned thin films based on geometrically-confined lateral crystal growth

**DOI:** 10.1038/ncomms15882

**Published:** 2017-07-10

**Authors:** Lynn Lee, Jangmi Baek, Kyung Sun Park, Yong-EunKoo Lee, Nabeen K. Shrestha, Myung M. Sung

**Affiliations:** 1 Department of Chemistry, Hanyang University, Seoul 04763, Korea; 2 Department of Energy and Materials Engineering, Dongguk University, Seoul 04620, Republic of Korea

## Abstract

We report a facile roll-printing method, geometrically confined lateral crystal growth, for the fabrication of large-scale, single-crystal CH_3_NH_3_PbI_3_ perovskite thin films. Geometrically confined lateral crystal growth is based on transfer of a perovskite ink solution via a patterned rolling mould to a heated substrate, where the solution crystallizes instantly with the immediate evaporation of the solvent. The striking feature of this method is that the instant crystallization of the feeding solution under geometrical confinement leads to the unidirectional lateral growth of single-crystal perovskites. Here, we fabricated single-crystal perovskites in the form of a patterned thin film (3 × 3 inch) with a high carrier mobility of 45.64 cm^2^ V^−1^ s^−1^. We also used these single-crystal perovskite thin films to construct solar cells with a lateral configuration. Their active-area power conversion efficiency shows a highest value of 4.83%, which exceeds the literature efficiency values of lateral perovskite solar cells.

## Abstract (web summary)

Wafer-scale deposition of uniform metal halide perovskite single-crystals is a step towards commercialisation. Using geometrically-confined lateral crystal growth, Lee *et al*., report patterned thin films of highly-aligned single-crystals and achieve lateral solar cells with efficiencies up to 4.83%.

Organic–inorganic hybrid perovskites, particularly CH_3_NH_3_PbX_3_ (X=Cl, Br, I), have received great attention due to their excellent optoelectronic properties at a low-cost production processes^1^,^2^,^3^,^4^,^5^,^6^,^7^,^8^,^9^,^10^. There have been extensive investigations on their use as promising materials for various optoelectronic devices such as solar cells, light-emitting diodes, lasers and photodetectors^11^,^12^,^13^,^14^,^15^,^16^,^17^,^18^,^19^. Most of these applications require perovskites in a thin film form; these are typically fabricated by means of spin-coating or vapour deposition. Recently, scalable production processes such as spray coating, blade coating and slot-die coating were reported to fabricate large-area perovskite photovoltaic devices^20^,^21^,^22^,^23^,^24^,^25^,^26^. However, it is difficult for these methods to produce a large-scale uniform and appropriate compositional thin film for highly efficient optoelectronics. To achieve high performance in the perovskite-based devices, it is crucial to employ high-quality single-crystal perovskite thin films with minimum structural defects, such as dislocations and grain boundaries. In principle, perovskite films with larger grains have lower densities of grain boundaries; therefore, their optoelectrical properties can be closer to those of single-crystal perovskites^27^,^28^,^29^. Recent efforts to obtain high-quality crystals have used a hot-casting technique to produce millimetre-scale grains of perovskites using a heated substrate to accelerate the growth of crystals in the presence of a solvent^4^,^30^. A study related to this method demonstrated that the crystal growth rate and the grain size are increased with increasing temperature, suggesting that instant crystallization is necessary to increase the grain size^31^.

Lateral crystal growth via epitaxial lateral overgrowth (ELO) is a common method that has traditionally been used to produce high-quality single-crystal semiconductor thin films, in which the vertical crystal growth is restricted by either kinetic control or geometrical confinement^32^,^33^. Recently, high-quality aligned single crystals of GaN were fabricated using evolutionary selection selective area growth^34^,^35^. During the early stage of this method, seed crystals with many misaligned grains grow in the channel patterned on an amorphous substrate, where the nucleus growth is restricted only in the open direction of the channels. During the next crystal propagation stage, lateral growth with geometrical confinement effectively filters out misaligned grains of the nucleus. After evolutionary selection of the crystal lattice orientation with the fastest growth rate, one crystal will dominate the channel space. These approaches suggest that geometrically confined lateral crystal growth during the crystal propagation step is necessary to fabricate defect-free single crystals.

Here, inspired by the hot-casting technique and lateral crystal growth, we developed a one-step printing method to fabricate single-crystal CH_3_NH_3_PbI_3_ perovskite thin films on wafer-scale substrates. The proposed method involves transfer of a perovskite ink solution via a channel-patterned rolling mould to the heated substrate to induce instant crystallization and lateral crystal growth under geometrical confinement. Thus, we obtained highly aligned single-crystal perovskites in the form of patterned thin films. On the basis of this growth mechanism, our fabrication approach is referred to as the geometrically confined lateral crystal growth (GC-LCG) method.

## Results

### Fabrication and observation of perovskite thin films

**Figure 1:**
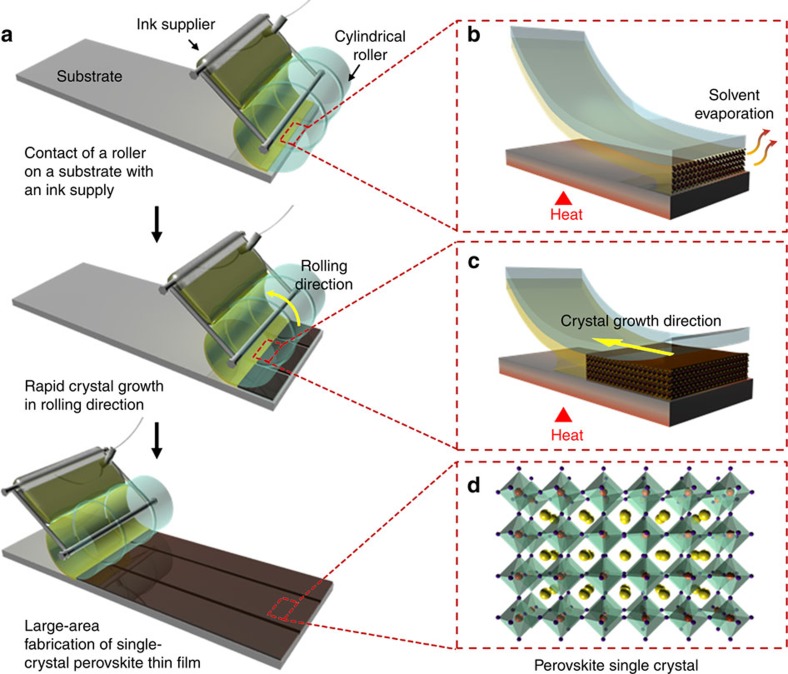
Fabrication of single-crystal perovskite patterned thin films. (**a**) Schematic of the manufacturing procedure for single-crystal perovskite thin films using geometrically confined lateral crystal growth (GC-LCG) with a rolling mould. The perovskite ink solution is geometrically confined between the mould and the substrate; thus, crystal growth is restricted in one direction. By rolling the mould and continuously supplying ink to the substrate, large-area single-crystal perovskite patterned thin films grow along the rolling direction. (**b**) Schematic illustration of the rapid solvent evaporation occurring at the open end of the channel. (**c**) Schematic illustration of the geometrically confined crystal growth along the channels of the mould. (**d**) Crystal structure of the single-crystal CH_3_NH_3_PbI_3_ perovskite thin films (yellow sphere: CH_3_NH_3_^+^, red sphere: Pb^2+^, violet sphere: I^−^).

Figure 1a illustrates the procedure used to fabricate patterned thin films of single-crystal perovskites with the GC-LCG process. A cylindrical metal roller is wrapped with a flexible poly(dimethylsiloxane) (PDMS) mould. The mould has a periodic array of wide and shallow channels (10 μm wide and 200 nm deep) with narrow spaces (400 nm wide) between the channels. First, the roller mounted with an ink supplier filled with the CH_3_NH_3_PbI_3_ perovskite precursor solution is placed in contact with the preheated SiO_2_ substrate (180 °C). Then, the perovskite ink solution is crystallized instantly at the open end of the channels with immediate solvent evaporation caused by the hot substrate, as illustrated in Fig. 1b. With an optimal mould rolling speed (1 mm s^−1^), the seed crystals can grow rapidly on the substrate via the instant crystallization. Here, crystal growth in the vertical direction is restricted because the deposited ink solution is vertically confined between the substrate and the shallow channels of the mould. Figure 1c describes the GC-LCG of the perovskite ink solution that occurs as the mould rolls. Lateral crystal growth under geometrical confinement effectively prevents misaligned growth of the seed crystals. Single-crystal CH_3_NH_3_PbI_3_ perovskite thin films with a tetragonal crystal structure are successfully fabricated by the GC-LCG process (Fig. 1d). The striking feature of this method is the ability to accurately control the crystal growth direction by taking advantage of the instant crystallization and geometrical confinement enabled by the roll-printing process.

**Figure 2:**
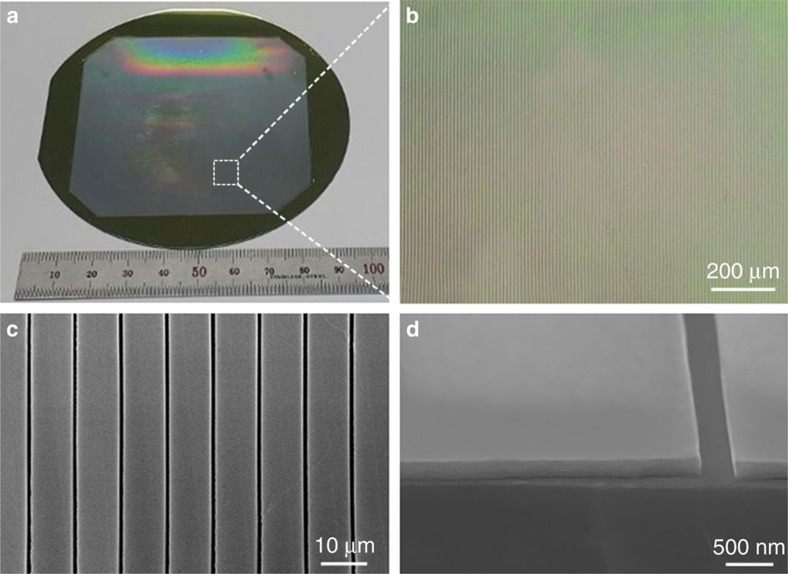
Wafer-scale single-crystal perovskite thin films. (**a**) Photographic image of the 3 × 3 inch perovskite thin films fabricated on a 4-inch, thermally oxidized Si substrate by the geometrically confined lateral crystal growth (GC-LCG) process. (**b**) Optical microscopic image of the perfectly aligned perovskite patterned thin films. (**c**) Scanning electron microscopic (SEM) image of single-crystal perovskite patterned thin film consisting of 10-μm-wide strips with 400-nm-wide spacing. (**d**) Cross-sectional SEM image of 200-nm-thick perovskite single-crystal strips, showing sharp edges and smooth morphology.

To demonstrate large-area crystal growth in a controlled direction using the GC-LCG method, a 3-inch-wide rolling mould was used to fabricate a patterned thin film. As shown in Fig. 2a, a patterned perovskite thin film (3 × 3 inch) was obtained on a 4-inch Si wafer using a line-patterned mould containing channels that were 10 μm wide and 200 nm deep with 400-nm-wide spacing. The optical microscopic (OM) image of the film shows a highly controlled unidirectional array of parallel strips (Fig. 2b). The detailed morphology of the films, obtained using scanning electron microscopy (SEM), demonstrates that all of the arrays have identical widths with equally narrow spaces between them (Fig. 2c). The area occupied by the gaps is less than 4% of the entire area of the patterned thin film. The dimensions of the dense and compact perovskite strips are in good accord with those of the channels in the mould. Two adjacent perovskite strips have a clearly defined space between them and sharp edges (Fig. 2d). Furthermore, the GC-LCG method can fabricate nanometre-sized patterns by simply using the nanoscale channel patterned moulds. Nanoscale patterned perovskite thin films were fabricated with smooth surface morphologies (Supplementary Fig. 1). It should be noted that under the optimized conditions, unidirectional single-crystal perovskite thin films with tunable pattern dimensions can be grown in the direction of rolling; hence the length of the film is determined by the distance travelled by the rolling mould. In contrast to the typical ELO method^32^,^33^, the crystallinity of the film grown by GC-LCG is independent to the epitaxy of the surface.

### Crystallographic properties of single-crystal perovskite thin film

**Figure 3:**
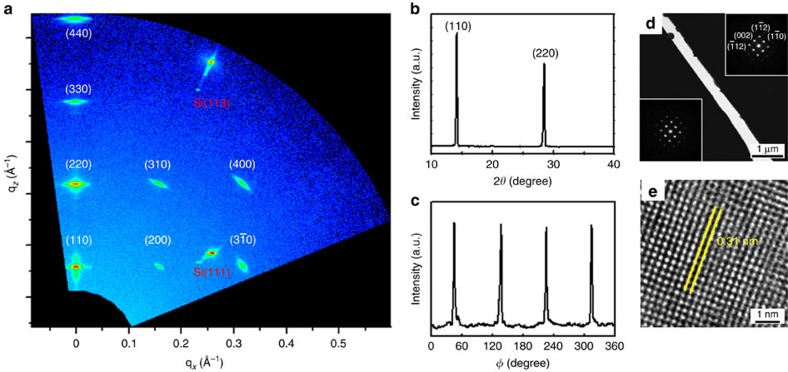
Crystallographic properties of single-crystal perovskite patterned thin films. (**a**) Two-dimensional X-ray diffraction (2D XRD) image of single-crystal perovskite patterned thin films prepared by the geometrically confined lateral crystal growth (GC-LCG) process using a line-patterned mould consisting of 10-μm-wide and 200-nm-thick strips with 400-nm-wide spacing. (**b**) Out-of-plane XRD scan of the single-crystal perovskite patterned thin film. Diffraction peaks are consistent with the diffraction spots along the q_z_ line obtained by 2D XRD. (**c**) In-plane *ϕ* scan of single-crystal perovskite patterned thin film obtained at the fixed 2θ angle of (002) plane. The four sharp peaks demonstrate the four-fold symmetry of tetragonal perovskite. (**d**) Transmission electron microscopic (TEM) image of single-crystal perovskite patterned thin films. Black areas indicate perovskite crystals, and the white vacancy is the space between crystals. Identical selected area electron diffraction patterns (insets) of each crystal show aligned crystals in the [001] direction. (**e**) High-resolution TEM image of a perovskite single crystal. The distance between yellow lines represents the d-spacing of the (004) lattice plane.

To investigate the crystallinity of the perovskite thin film prepared by GC-LCG, X-ray diffraction and transmission electron microscopy (TEM) were employed. Two-dimensional (2D) X-ray diffraction was performed to examine the crystal plane orientation of the film. The patterned CH_3_NH_3_PbI_3_ thin film was placed to parallel the incident beam propagation direction and analysed using a 2D detector (Supplementary Fig. 2). Figure 3a shows the 2D X-ray diffraction image, displaying the reciprocal space map (RSM) with the scattering vector (*q*) that is the inverse of the distance (*d*) between the adjacent lattice planes (*q*=1/*d*). The sharp and strong reflection spots indicate that the film created by GC-LCG has a single-crystal nature compared to the several diffraction rings from the RSM of polycrystalline thin film (Supplementary Fig. 3a). The reflection spots in the out-of-plane direction of *q*_z_ (that is, surface normal) observed at 0.16, 0.32, 0.48 and 0.64 Å^−1^ correspond to the (110), (220), (330) and (440) planes of the CH_3_NH_3_PbI_3_ crystal, respectively. These reflection peaks indicate that the [110] axis orientation of the patterned thin film is perpendicular to the substrate surface. Similarly, the other clear spots can be assigned to the (200), (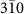), (310) and (400) planes of the CH_3_NH_3_PbI_3_ perovskite. Because the reciprocal mapping plane is perpendicular to the incident beam direction, which is parallel to the growth direction of crystals, one can infer that the zone axis is the unidirectional (001) axis. Thus, this axis is the preferential growth direction of the perovskite crystals along the channels in the GC-LCG process (details provided in Supplementary Fig. 2).

The out-of-plane X-ray diffraction data of the single-crystal thin film show diffraction peaks at only 14.1° and 28.5°, corresponding to the (110) and (220) planes, respectively, (Fig. 3b). This is identical to the diffraction spots on the *q*_z_ line in the RSM. This result confirms that the highly oriented perovskite film is primarily deposited in the (110) direction (that is, surface normal). The four sharp peaks appearing every 90° obtained by the in-plane phi scan demonstrate the highly ordered fourfold symmetry of the tetragonal perovskite crystal of CH_3_NH_3_PbI_3_ (Fig. 3c). The X-ray diffraction scan of the polycrystalline thin film presents additional diffraction peaks when compared to that of single-crystal perovskite thin films, meaning that other misaligned crystal planes are grown along the surface normal direction (Supplementary Fig. 3b). Figure 3d shows a TEM image of the patterned perovskite film; the insets are the corresponding selective area electron diffraction (SAED) pattern images from the two adjacent strips. More SAED patterns and cross-sectional TEM images are obtained from a plenty of samples, various locations for each sample (Supplementary Figs 4 and 5). The perfect single-crystal nature of the perovskite film is confirmed by the SAED patterns, exhibiting only reflections related to the tetragonal structure oriented along the [110] zone axis, that is, the (002) and (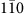) spots. These results also indicate that the single-crystal perovskite film grows laterally in the channels of the moulds along the [001] direction, which agree with the results obtained by X-ray diffraction. The single-crystal perovskite film was further investigated using high-resolution (HR) TEM. Figure 3e shows the crystal lattice image of the perovskite micro-strip with a lattice spacing of 3.1 Å, corresponding to the perovskite (004) plane. The nanoscale perovskite patterns also demonstrate identical crystallinity with the crystal structure of the 10-μm-wide perovskite patterns (Supplementary Fig. 6). The optical properties of single-crystal thin films were characterized using the ultraviolet-visible and photoluminescence (PL) spectroscopy, and time resolved photoluminescence (TRPL) (Supplementary Fig. 7). The elemental composition of the perovskite strip was analysed using an energy dispersive X-ray (EDX) analyser coupled to the TEM, which revealed the presence of nitrogen (N), lead (Pb) and iodine (I) at an atomic ratio of 1:1:3 (Supplementary Fig. 8). This ratio is close to the theoretical stoichiometry of N, Pb and I in CH_3_NH_3_PbI_3_ molecules; hence, these results strongly support the formation of the single-crystal perovskite thin film using the proposed GC-LCG method. This is a successful demonstration of the formation of unidirectional aligned strips of planar single-crystal perovskite thin films.

GC-LCG is a highly effective method that can be used to fabricate large-area single-crystal perovskite thin films; it is based on instant crystallization followed by lateral crystal growth under geometrical confinement. Initially, seed crystals with a [110] preferred orientation^4^,^30^,^36^ along the vertical direction are produced at the open end of the channels by instant crystallization. This lattice orientation of the seeds is the same as the crystal direction of the perovskite thin films grown via hot casting. Then, the vertical confinement provided by the patterned mould restricts further growth in the vertical direction; thus, the perovskite crystals only grow in the lateral direction. While subsequent crystal growth on the seeds is allowed along the channel direction, other misaligned seed grains are filtered by lateral confinement. In this way, the most dominant crystal with a (002) lattice plane is formed in a single crystal. In this method, instant crystallization plays an important role in inducing the formation of seed crystals, and leading to subsequent fast crystal growth. A geometrically confined environment is another key factor that allows the dominant crystal growth from the seed crystals with a specified orientation in the single-crystal perovskites. The temperature of the substrate is maintained at 180 °C (that is, higher than the solvent boiling point) to enable rapid solvent evaporation, thereby sequentially facilitating instant crystallization process (Supplementary Fig. 9). It was determined that the optimal rolling speed was in the range of 0.2–3 mm s^−1^; other rolling speeds yielded polycrystalline perovskite thin films. Slower rolling speeds lead to a longer processing time, which results in heat decomposition of the perovskite film (Supplementary Fig. 10a). Faster rolling of the mould produces branched perovskite arrays due to insufficient time for crystallization in the confined channels (Supplementary Fig. 10b).

The geometrical confinement is a critical factor in the formation of highly aligned single-crystal perovskite thin films. When the perovskite thin films are prepared under the same experimental conditions with the exception of geometrical confinement (that is, the ink is spread by a flat-surface roller), the crystal grains extend in multiple directions and produce a branched polycrystalline form (Supplementary Fig. 11). The typical mould in this study contains wide, shallow channels (10 μm wide and 200 nm deep) for geometrical confinement. Moulds containing channels with different thicknesses (500 nm, and 1 μm with an identical channel width of 10 μm) are also used to successfully fabricate perovskite thin films by the GC-LCG process (Supplementary Fig. 12). The surface morphology of each 200- and 500-nm-thick film is smooth, while the surface of the 1-μm-thick film is rough and agglomerated. Their crystallinities are investigated by X-ray diffraction; these results show a single crystalline nature in both the 200- and 500-nm-thick films and polycrystallinity in the 1-μm-thick film (Supplementary Fig. 13). This demonstrates that the crystal growth behaviour significantly relies on the vertical confinement, which is determined by the channel depth.

### Electrical properties of single-crystal perovskite thin film

**Figure 4:**
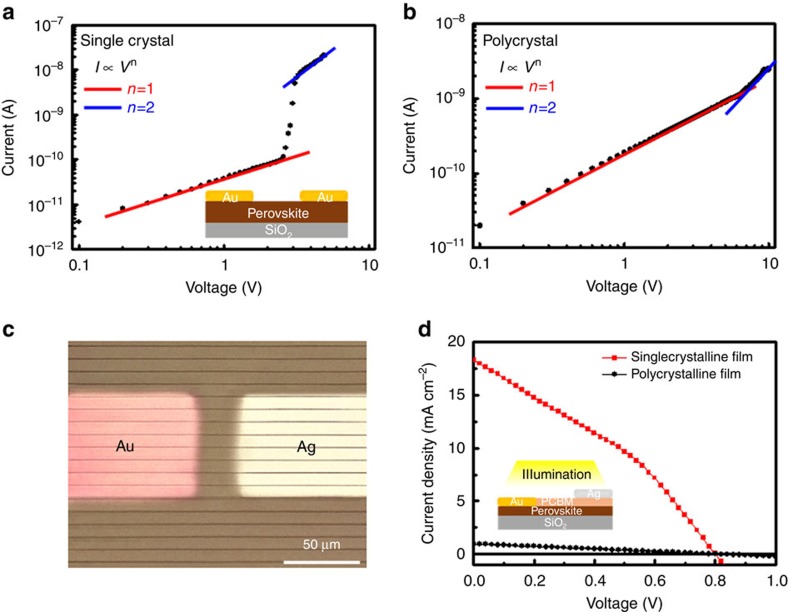
Electrical properties of single-crystal perovskite thin films. (**a**) *I–V* curve of 200-nm-thick single-crystal perovskite patterned thin film prepared by the geometrically confined lateral crystal growth (GC-LCG) process. The inset shows a schematic of the gap-type device used for space-charge-limited current measurements. (**b**) *I–V* curve of the 200-nm-thick polycrystalline perovskite thin film prepared by spin-coating. (**c**) An optical microscopic image of a single-crystal perovskite lateral perovskite solar cell with metal electrodes. The light brown and dark brown lines indicate perovskite single crystals and spaces, respectively. (**d**) *J–V* curves of single crystalline (red) and polycrystalline (black) perovskite thin film lateral perovskite solar cells. The inset shows a schematic device structure of a lateral perovskite solar cell and the direction of illumination.

The carrier mobility of the single-crystal perovskite film is evaluated via space-charge-limited current (SCLC) measurements. A gap-type device, which is preferred for SCLC measurements of thin film semiconductors, was fabricated using 200-nm-thick single-crystal CH_3_NH_3_PbI_3_ with two 100-μm-gap Au electrodes (Fig. 4a, inset)^37^,^38^. Figure 4a presents the dark current–voltage (*I–V*) curve of the single-crystal perovskite. Typically, a SCLC *I–V* curve shows two different regimes depending on applied voltages, including transition from ohmic regime (*I*∝*V*) at low voltages to SCLC regime (*I*∝*V*^2^) at high voltages. The current from SCLC region is mainly driven by the injected charge carriers from the electrode and therefore the current depends only on the mobility. From this *I–V* curve, we can derive the carrier mobility for gap-type geometry by the Geurst’s SCLC model^37^,^38^:


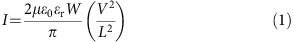


where *μ* is the carrier mobility, *ε*_0_ is the vacuum permittivity and *ε*_r_ is relative dielectric constant of perovskite, 32 (ref. 7). *W* and *L* are the device width and length, respectively. For our single-crystal perovskite films, the hole mobility of 45.64 cm^2^ V^−1^ s^−1^ was obtained from quadratic region over 3.0 V, which is comparable to the values from previous studies of bulk single-crystal perovskite^1^,^5^,^7^. The typical SCLC regime for the polycrystalline perovskite thin film was also observed, resulting in the mobility of 0.2512, cm^2^ V^−1^ s^−1^ (Fig. 4b). Due to the high carrier mobility, the single-crystal thin film is ideal for facilitating the improved performance of optoelectronic devices. As a proof-of-concept application, a perovskite solar cell with a lateral configuration was prepared by sequentially depositing an Au electrode, PCBM layer, and Ag electrode onto the perovskite thin film (Fig. 4c). Poling was carried out before the power conversion efficiency (PCE) measurement to form p-i-n structure in the perovskite^39^,^40^. During the poling procedure, the external potential field by DC power supplier is applied to the perovskite solar cell devices so as to induce the ion migration inside of the perovskite and thereby to derive the p-i-n structure of the perovskite layer. The voltage was 13 V in 33-μm channel length, which was calculated from the poling field of 0.39 V μm^−1^. The light intensity is 100 mW cm^−2^, AM 1.5G and the active area is 30 × 77.2 μm^2^. The best active-area PCE of the single-crystal-based devices is 4.83% with a short-circuit current density (*J*_sc_) of 18.33 mA cm^−2^, an open-circuit voltage (*V*_oc_) of 0.801 V, and a fill factor (FF) of 0.329 as revealed by the current density–voltage (*J–V)* curve in Fig. 4d (red line). This is by far the highest active-area PCE value ever reported for a lateral perovskite solar cell. An average efficiency of 4.14% is recorded from 100 solar cell devices with very low distribution of efficiency (ca.±0.7%, Supplementary Fig. 14). In comparison, a lateral perovskite solar cell using a polycrystalline perovskite film fabricated in the same way provides a maximum efficiency of 0.194% with a *J*_sc_ of 0.974 mA cm^−2^, a *V*_oc_ of 0.803 V and a FF of 0.248 (Fig. 4d, dark line). Due to the higher mobility in the single-crystal perovskite compared to the polycrystalline one, outstanding PCEs are observed in the single-crystal perovskite-based devices. External quantum efficiency (EQE) is up to 80% in the broad spectral range of 450–750 nm (Supplementary Fig. 15). The PCE measurements suggest that single-crystal perovskite thin films make good use of the lateral configuration, enabling the fabrication of flexible devices and one-step series and shunt integration to increase the voltage and current of devices.

## Discussion

In summary, a facile one-step roll-imprinting method for growing wafer-scale perovskite thin films with controllable crystal direction has been developed. This process enables the fabrication of highly oriented single-crystal thin films with high carrier mobility. In addition, the crystal orientation of these films is determined by the rolling direction, indicating that the proposed method enables perovskites to be used in integrated optoelectronic devices. The concept of this method is universal and can be expanded to fabricate various semiconductor and polymer materials, regardless of the epitaxy of the substrates.

## Methods

### Preparation of perovskite ink solution

CH_3_NH_3_I (99%, Dyesol), PbI_2_ (99%, Sigma-Aldrich Inc.), and *N,N*-dimethylformamide (DMF, anhydrous 99%, Sigma-Aldrich Inc.) were used without further purification. Perovskite ink solutions with concentrations ranging from 20 to 50 wt% were prepared for single-crystal perovskite fabrication by dissolving an equimolar ratio (1:1) of CH_3_NH_3_I and PbI_2_ in DMF. These solutions were stirred overnight at 60 °C in an argon-filled glovebox.

### Preparation of mould and substrate

First, patterned Si masters with various dimensional line patterns were created by e-beam lithography. The PDMS precursor (Sylgard 184, Dow Corning) was prepared by mixing a 10:1 ratio of PDMS precursor to the cross-linking agent. This mixture was cast onto the patterned Si masters. After curing at 70 °C for 1 h, the PDMS moulds were peeled away from the masters. The PDMS moulds were mainly used to fabricate perovskite thin films in this study. A hard mould was also used to fabricate the 100-nm-wide patterned perovskite thin films. Trimethylolpropane propoxylate triacrylate (TPT, Sigma-Aldrich Inc.) and 2-hydroxy-2-methylpropiophenone (97%, Sigma-Aldrich Inc.) were mixed at a 10:1 ratio and cast onto the patterned Si masters. This was followed by Ultraviolet curing for 15 min. These flexible thin moulds were wrapped on cylindrical stainless-steel rollers, which were used as rolling moulds. The substrates used in this study were heavily doped, *p*-type Si wafers (Namkang Hi-Tech Inc.) with a 300-nm-thick thermally grown SiO_2_ layer on the surface. The substrates were washed with ethanol, dried in a stream of N_2_, and ultraviolet/ozone treated for 5 min to remove contaminants.

### Growth of single-crystal perovskite patterned thin films

Single-crystal perovskite patterned thin films were fabricated on SiO_2_/Si substrates using rolling moulds with various line-pattern dimensions. Moulds with five different sets of channel dimensions (that is, depth/width/spacing) were used to form crystal films: 200 nm/100 nm/100 nm, 200 nm/600 nm/100 nm, 200 nm/10 μm/400 nm, 500 nm/10 μm/400 nm and 1 μm/10 μm/400 nm. Ink concentrations of 20 and 50 wt% were used to fabricate nanoscale and microscale perovskite thin films, respectively. A homemade cylindrical rolling mould, consisting of a continuous ink supplier, was designed to deposit the perovskite thin films. For the fabrication of thin films, the mould was placed on a substrate preheated to 180 °C and then rolled over the substrate in one direction at a constant rolling speed between 200 μm s^−1^ and 3 mm s^−1^. Under optimized conditions with a rolling speed of 1 mm s^−1^, 3-inch wafer-scale single-crystal perovskite patterned thin films (that is, 200 nm/10 μm/400 nm) were achieved on 4-inch SiO_2_ wafers. After rolling was complete, the substrates were cooled immediately to avoid the possibility of thermal decomposition. All experiments were carried out under ambient conditions.

### Structural characterization

Perovskite thin films were characterized using an optical microscope (ICS-305B, Sometech) and a scanning electron microscope (SEM, Hitachi S4800) with an accelerating voltage of 15 kV. The optical properties were measured using an Ultraviolet-Visible spectrophotometer (Varian Cary 5000, Agilent Technologies) and a fluorescence spectrometer (FLSP920, Edinburgh Instruments). The TRPL was measured using a commercial time correlated single photon counting (TCSPC) system (FluoTim 200, PicoQuant). The crystallinity and elemental analysis of the thin films were determined via SAED and EDX spectrometry (AZtec software, Oxford instruments) with a transmission electron microscope (TEM, JEM 2100F, JEOL.) at an accelerating voltage of 200 kV. For preparing the cross-sectional TEM sample, the focused ion beam (FIB) process was applied (Nova 600, Nanolab). The cross-sectional TEM samples were measured using Cs-corrected TEM (JEM-ARM200F, JEOL). The large-area crystallinity was measured by an X-ray diffractometer (D/MAX-2500/PC, Rigaku) using a Cu Kα source (*λ*=1.54 Å) operated at 2.4 kW and a HR X-ray diffractometer (Smartlab, Rigaku) with a 2D detector (PILATUS 100K, Rigaku) using a Cu Kα source operating at 9 kW. For the in-plane phi scan, the 2*θ* angle was fixed at 14°. For the two-dimensional X-ray diffraction (2D XRD) measurements, the X-ray analysed area of the film was 5 × 5 mm. The range of measurement was 10°–55° in the 2*θ* direction and 0°–60° in the chi direction.

### Devices fabrication and characterization

Gap-type devices based on single-crystal and polycrystalline perovskite thin films were fabricated by depositing films on 300-nm-thick SiO_2_/Si (100) substrates. The rolling mould (200-nm-deep and 10-μm-wide channels) was used to fabricate single-crystal perovskite thin films. A polycrystalline perovskite thin film was deposited by spin-coating a perovskite ink solution with a concentration of 20 wt% on an SiO_2_ substrate at 3,000 r.p.m. for 60 s. The film was then annealed on a hotplate at 70 °C for 30 min. Subsequently, 200-nm-thick source and drain Au electrodes with a gap length of 100 μm were deposited thermally at a rate of 1 Å s^−1^. These gap-type devices were used for SCLC measurements to estimate the hole mobility in the perovskite films. To study the light-collecting properties of the perovskite films, lateral perovskite solar cells were fabricated by depositing single-crystal perovskite thin films using a rolling mould with 500-nm-deep and 10-μm-wide channels. A 200-nm-thick Au electrode was thermally deposited on the film through a metal mask. A 1 wt% PCBM solution in chlorobenzene was spin-coated on the film at 3,000 r.p.m. for 30 s to serve as an electron transport layer. Finally, a 200-nm-thick Ag electrode was thermally deposited through a mask at a distance of 33 μm from the predeposited Au electrode. During the measurement, the devices were covered by a mask with an area of 30 × 80 μm^2^. The active area in all measurement was defined as an area of 30 × 77.2 μm^2^ by considering the present of gaps between perovskite strips. A polycrystalline perovskite-based device was also prepared for comparison by spin-coating a film at 2,500 r.p.m. for 60 s using a precursor solution with a concentration of 40 wt%. Electrical poling was applied to the devices at an electric field of 0.39 V μm^−1^ for 60 s to induce a p-i-n junction^39^,^40^. The gap-type devices and solar cells were measured using a semiconductor parameter analyser (HP 4156C, Agilent Technologies) in ambient air conditions. The presented *J–V* curves of devices are the ones in reverse directional scan. The *J–V* curves in both reverse and forward directions under low light intensity are shown in Supplementary Fig. 16a. Note that the *J–V* curve in the reverse direction is determined by scanning from open-circuit to short-circuit, while that in the forward direction is vice versa. The device reliability and stability data from different scan rates and overtime evolution are shown in Supplementary Figs 16b and 17. The photocurrent was measured with a continuous scanning rate of 100 mV s^−1^ with dwell time of 50 ms under 100 mW cm^−2^ illumination using a Halogen lamp-based standard solar simulator (PEC-L01, Peccell Technologies, Inc.). The light intensity was set to standard AM 1.5G condition. The spectral profile of the light source (Xenon lamp) is shown in Supplementary Fig. 18. The EQE spectrum of the lateral perovskite solar cell was obtained using an incident photon-to-electron conversion efficiency (IPCE) measurement system with the light spectrum of AM 1.5G (K3100 IQX, McScience Inc.).

### Data availability

The data sets generated during the current study are available from the corresponding authors.

## Additional information

**How to cite this article:** Lee, L. *et al*. Wafer-scale single-crystal perovskite patterned thin films based on geometrically-confined lateral crystal growth. *Nat. Commun.* **8**, 15882 doi: 10.1038/ncomms15882 (2017).

**Publisher’s note:** Springer Nature remains neutral with regard to jurisdictional claims in published maps and institutional affiliations.

## Supplementary Material

Supplementary Information

## References

[b1] Liu Y. . Two-inch-sized perovskite CH_3_NH_3_PbX_3_ (X = Cl, Br, I) crystals: growth and characterization. Adv. Mater. 27, 5176–5183 (2015). 26247401 10.1002/adma.201502597

[b2] Ha S. T. . Synthesis of organic–inorganic lead halide perovskite nanoplatelets: towards high-performance perovskite solar cells and optoelectronic devices. Adv. Opt. Mater. 2, 838–844 (2014).

[b3] Saidaminov M. I. . High-quality bulk hybrid perovskite single crystals within minutes by inverse temperature crystallization. Nat. Commun. 6, 7586 (2015). 26145157 10.1038/ncomms8586 PMC4544059

[b4] Nie W. . High-efficiency solution-processed perovskite solar cells with millimeter-scale grains. Science 347, 522–525 (2015). 25635093 10.1126/science.aaa0472

[b5] Shi D. . Low trap-state density and long carrier diffusion in organolead trihalide perovskite single crystals. Science 347, 519–522 (2015). 25635092 10.1126/science.aaa2725

[b6] Stranks S. D. . Electron-hole diffusion lengths exceeding 1 micrometer in an organometal trihalide perovskite absorber. Science 342, 341–344 (2013). 24136964 10.1126/science.1243982

[b7] Dong Q. . Electron-hole diffusion lengths > 175 μm in solution-grown CH3NH3PbI3 single crystals. Science 347, 967–970 (2015). 25636799 10.1126/science.aaa5760

[b8] Kojima A., Teshima K., Shirai Y. & Miyasaka T. Organometal halide perovskites as visible-light sensitizers for photovoltaic cells. J. Am. Chem. Soc. 131, 6050–6051 (2009). 19366264 10.1021/ja809598r

[b9] Kim H. S. . Lead iodide perovskite sensitized all-solid-state submicron thin film mesoscopic solar cell with efficiency exceeding 9%. Sci. Rep. 2, 591 (2012). 22912919 10.1038/srep00591 PMC3423636

[b10] Lee M. M., Teuscher J., Miyasaka T., Murakami T. N. & Snaith H. J. Efficient hybrid solar cells based on meso-superstructured organometal halide perovskites. Science 338, 643–647 (2012). 23042296 10.1126/science.1228604

[b11] Kazim S., Nazeeruddin M. K., Grätzel M. & Ahmad S. Perovskite as light harvester: a game changer in photovoltaics. Angew. Chem. Int. Ed. 53, 2812–2824 (2014). 10.1002/anie.201308719 24519832

[b12] Mei A. . A hole-conductor–free, fully printable mesoscopic perovskite solar cell with high stability. Science 345, 295–298 (2014). 25035487 10.1126/science.1254763

[b13] Peng W. . Solution-grown monocrystalline hybrid perovskite films for hole-transporter-free solar cells. Adv. Mater. 28, 3383–3390 (2016). 26931100 10.1002/adma.201506292

[b14] Zhou H. . Interface engineering of highly efficient perovskite solar cells. Science 345, 542–546 (2014). 25082698 10.1126/science.1254050

[b15] Zhu H. . Lead halide perovskite nanowire lasers with low lasing thresholds and high quality factors. Nat. Mater. 14, 636–642 (2015). 25849532 10.1038/nmat4271

[b16] Tan Z.-K. . Bright light-emitting diodes based on organometal halide perovskite. Nat. Nano 9, 687–692 (2014). 10.1038/nnano.2014.149 25086602

[b17] Fang Y., Dong Q., Shao Y., Yuan Y. & Huang J. Highly narrowband perovskite single-crystal photodetectors enabled by surface-charge recombination. Nat. Photon. 9, 679–686 (2015).

[b18] Yakunin S. . Detection of X-ray photons by solution-processed lead halide perovskites. Nat. Photon. 9, 444–449 (2015). 10.1038/nphoton.2015.82 PMC5444515 28553368

[b19] Saidaminov M. I. . Planar-integrated single-crystalline perovskite photodetectors. Nat. Commun. 6, 1–7 (2015). 10.1038/ncomms9724 PMC4667636 26548941

[b20] Chen Y., He M., Peng J., Sun Y. & Liang Z. Structure and growth control of organic–inorganic halide perovskites for optoelectronics: from polycrystalline films to single crystals. Adv. Sci. 3, 1–21 (2016). 10.1002/advs.201500392 PMC5069589 27812463

[b21] Tait J. G. . Rapid composition screening for perovskite photovoltaics via concurrently pumped ultrasonic spray coating. J. Mater. Chem. C 4, 3792–3797 (2016).

[b22] Yang Z. . High-performance fully printable perovskite solar cells via blade-coating technique under the ambient condition. Adv. Energy Mater. 5, 1–6 (2015).26190957

[b23] Kim J. H., Williams S. T., Cho N., Chueh C.-C. & Jen A. K. Y. Enhanced environmental stability of planar heterojunction perovskite solar cells based on blade-coating. Adv. Energy Mater. 5, 1–6 (2015).26190957

[b24] Cho N. . Pure crystal orientation and anisotropic charge transport in large-area hybrid perovskite films. Nat. Commun. 7, 1–11 (2016). 10.1038/ncomms13407 PMC5109592 27830694

[b25] Deng Y. . Scalable fabrication of efficient organolead trihalide perovskite solar cells with doctor-bladed active layers. Energy Environ. Sci. 8, 1544–1550 (2015).

[b26] Hwang K. . Toward large scale roll-to-roll production of fully printed perovskite solar cells. Adv. Mater. 27, 1241–1247 (2015). 25581092 10.1002/adma.201404598

[b27] Mastroianni S. . Analysing the effect of crystal size and structure in highly efficient CH_3_NH_3_PbI_3_ perovskite solar cells by spatially resolved photo- and electroluminescence imaging. Nanoscale 7, 19653–19662 (2015). 26548804 10.1039/c5nr05308k

[b28] Kim H. D., Ohkita H., Benten H. & Ito S. Photovoltaic performance of perovskite solar cells with different grain sizes. Adv. Mater. 28, 917–922 (2016). 26639125 10.1002/adma.201504144

[b29] Chen J. . Origin of the high performance of perovskite solar cells with large grains. Appl. Phys. Lett. 108, 053302 (2016).

[b30] Zheng Y. C. . Thermal-induced volmer–weber growth behavior for planar heterojunction perovskites solar cells. Chem. Mater. 27, 5116–5121 (2015).

[b31] Dualeh A. . Effect of annealing temperature on film morphology of organic–inorganic hybrid pervoskite solid-state solar cells. Adv. Funct. Mater. 24, 3250–3258 (2014).

[b32] Müller G., Métois J.-J. & Rudolph P. Crystal Growth-from Fundamentals to Technology Elsevier (2004).

[b33] Herman M. A., Richter W. & Sitter H. Epitaxy: Physical Principles and Technical Implementation Springer Science & Business Media (2013).

[b34] Kachkanov V. . Structural dynamics of gan microcrystals in evolutionary selection selective area growth probed by X-ray microdiffraction. Sci. Rep. 4, 1–6 (2014). 10.1038/srep04651 PMC3983619 24722064

[b35] Leung B., Song J., Zhang Y. & Han J. Evolutionary selection growth: towards template-insensitive preparation of single-crystal layers. Adv. Mater. 25, 1285–1289 (2013). 23233293 10.1002/adma.201204047

[b36] Yang B. . Perovskite solar cells with near 100% internal quantum efficiency based on large single crystalline grains and vertical bulk heterojunctions. J. Am. Chem. Soc. 137, 9210–9213 (2015). 26156790 10.1021/jacs.5b03144

[b37] Zuleeg R. & Knoll P. Space‐charge‐limited currents in heteroepitaxial films of silicon grown on sapphire. Appl. Phys. Lett. 11, 183–185 (1967).

[b38] Geurst J. A. Theory of space-charge-limited currents in thin semiconductor layers. Phys. Status Solidi 15, 107–118 (1966).

[b39] Xiao Z. . Giant switchable photovoltaic effect in organometal trihalide perovskite devices. Nat. Mater. 14, 193–198 (2015). 25485985 10.1038/nmat4150

[b40] Dong Q. . Lateral-structure single-crystal hybrid perovskite solar cells via piezoelectric poling. Adv. Mater. 28, 2816–2821 (2016). 26836224 10.1002/adma.201505244

