# Supplementary material for: Wafer-scale single-crystal perovskite patterned thin films based on geometrically-confined lateral crystal growth
Source: Nat Commun. 2017 Jul 10;8:15882. doi: 10.1038/ncomms15882 (PMC5508126; doi:10.1038/ncomms15882)
Supplement: Supplementary Information [file ncomms15882-s1.pdf]

Title of file for HTML: Supplementary Information
Description: Supplementary Figures and Supplementary References

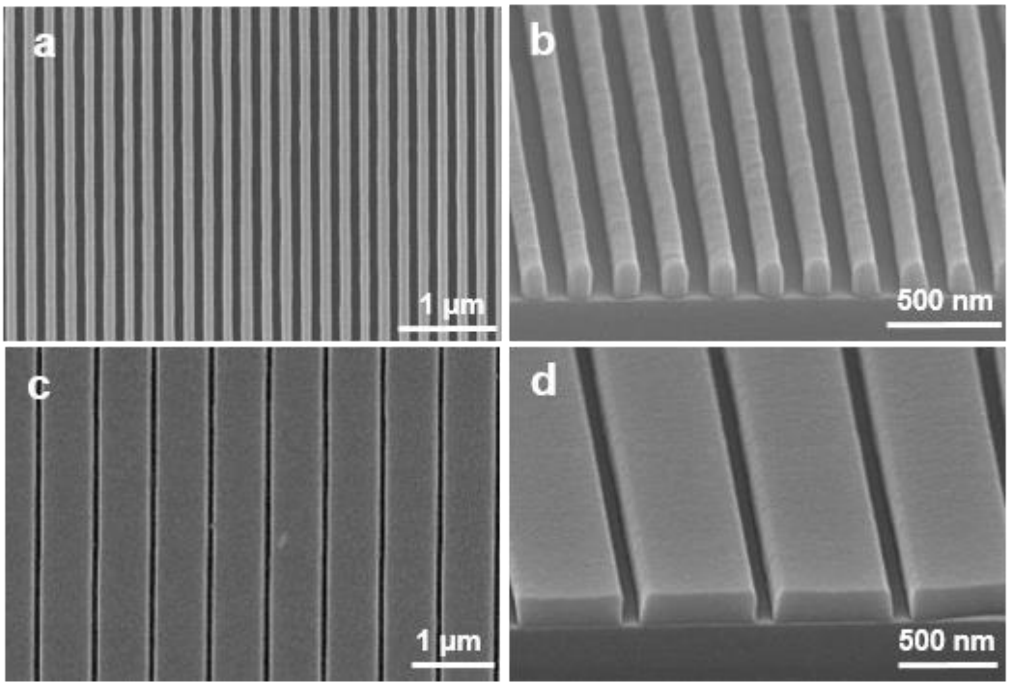

Supplementary Figure 1. SEM images of perovskite single-crystal patterned thin film with nanoscale width. (**a**) The perovskite thin film consisting of 100-nm-wide strips aligned in one direction. (**b**) Cross-sectional SEM image of the perovskite patterned thin film consisting of nanoscale periodic strips. (**c**) The perovskite thin film with 600-nm-wide strips aligned in one direction. (**d**) Cross-sectional SEM image of perovskite patterned thin film consisting of the periodic strips. Perovskite thin films consisting of nanoscale strips were successfully fabricated using the GC-LCG process; these films had smooth surface morphologies.

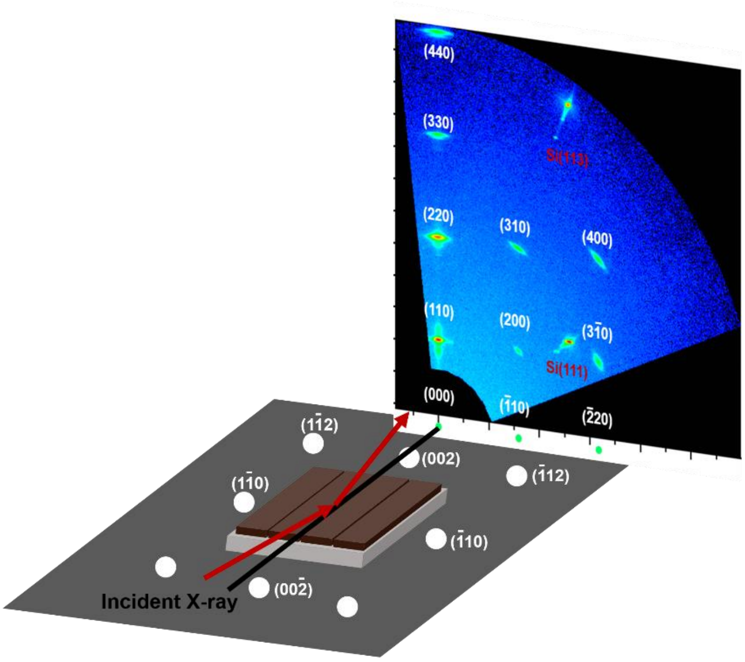

Supplementary Figure 2. Schematic illustration of the 2D XRD characterization setup used to observe the crystal orientation of single-crystal $CH_3NH_3PbI_3$ perovskite thin films. The incident X-ray beam is parallel to the crystal growth direction; thus, the obtained reciprocal space map (chi angle between 0 and 60°) provides lattice plane information that is perpendicular to the substrate. The schematic SAED pattern illustrates the orientation of crystals as seen in the [110] zone axis, corresponding to the q_z diffraction spots in RSM. By combining the results from the two different crystal characterizations, the preferential crystal growth in the channels with the GC-LCG process is inferred to be in the [001] direction.

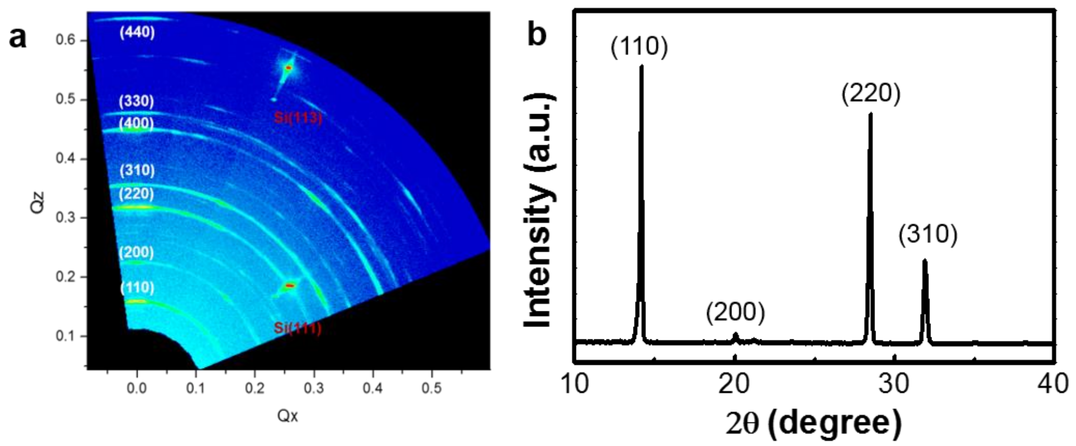

Supplementary Figure 3. Crystallographic properties of the polycrystalline perovskite thin film. (a) A representative 2D XRD map of a spin-coated perovskite thin film. Several diffraction rings are shown, indicating polycrystalline nature of a spin-coated perovskite thin film. (b) A representative XRD scan of a spin-coated perovskite thin film. Distinct (200) and (310) peaks are additionally observed when compared to the XRD scan of single-crystal perovskite thin films that reveals only {110} peak.

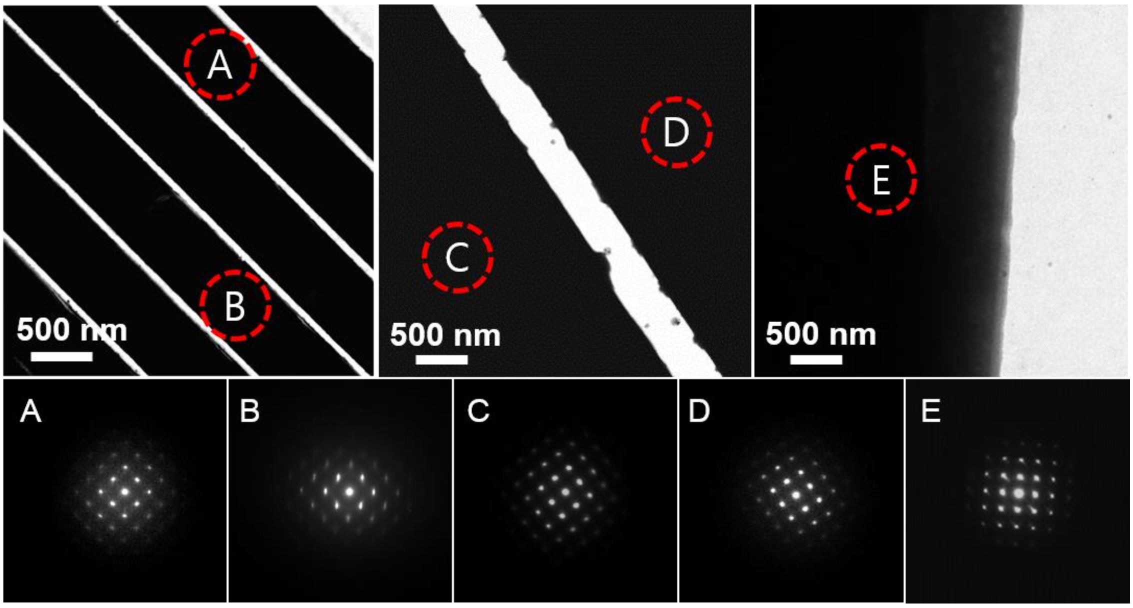

Supplementary Figure 4. The top-view TEM images of various perovskite patterned thin films and their corresponding SAED patterns from selected areas (A - E). Identical SAED patterns of each crystal show highly aligned crystals grown in the [001] direction.

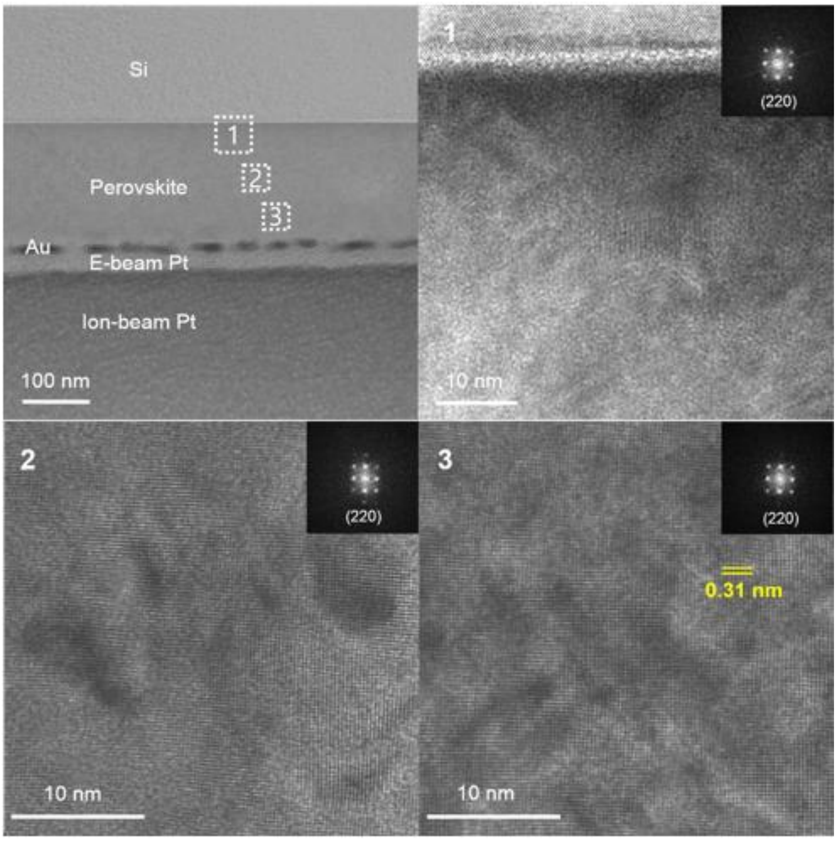

Supplementary Figure 5. The cross-sectional TEM images of the patterned perovskite thin film. A low-resolution TEM image of the perovskite thin film shows flat and smooth cross section of the perovskite without any stacked crystals. The selected HR-TEM images and its corresponding fast Fourier transform (FFT) patterns show the constant lattice fringes, resulting in single crystalline nature of our perovskite thin film. The FFT of image 1 presents the selected area of perovskite crystal. The d-spacing is around 0.31 nm which is identical to the (220) lattice plane, corresponded to the diffraction spot from the 2D XRD.

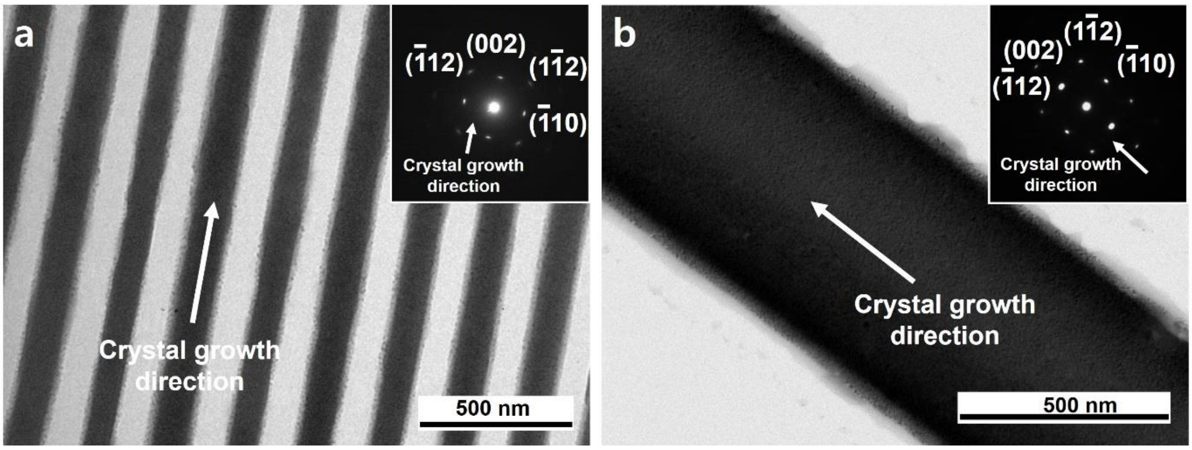

Supplementary Figure 6. (**a**), (**b**) TEM images of (**a**) 100-nm-wide and (**b**) 600-nm-wide perovskite crystals. Crystals with nanoscale widths are successfully achieved by the GC-LCG process, and the crystallinity of each crystal is represented in the inset SAED pattern. The assigned lattice spacing of the tetragonal perovskite structure indicates the single crystalline nature of the perovskite crystals. The results also show that the axis of growth for each of the perovskite crystals is in the same [001] direction irrespective of the channel width.

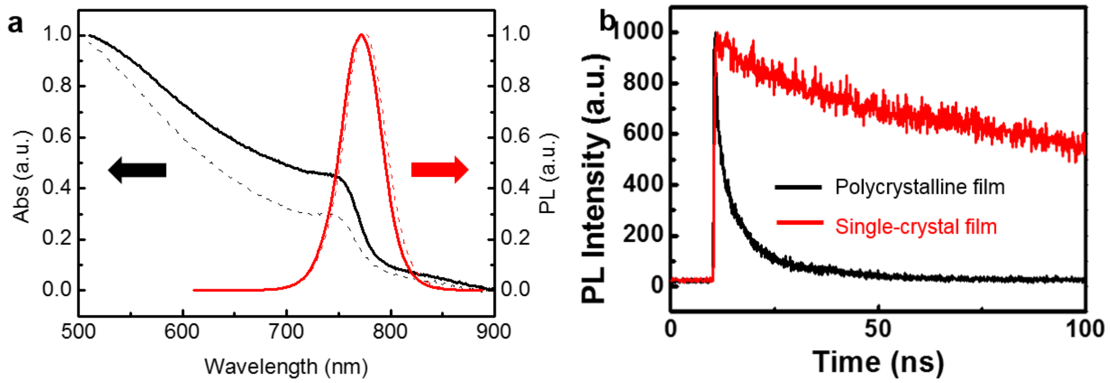

Supplementary Figure 7. Optical properties of a 200-nm-high single-crystal perovskite patterned thin film. (**a**) UV-Vis absorption and PL spectrum. A sharper absorption edge (black solid line) and a blue shift in the PL peak (red solid line) are observed for the patterned single-crystal perovskite thin film compared to those for the polycrystalline thin film (each dashed black and red line) [1, 2]. (**b**) TRPL of the single-crystal and polycrystalline perovskite patterned thin films. The PL decay of the patterned single-crystal film is much longer than that of hot-casted polycrystalline film [3, 4].

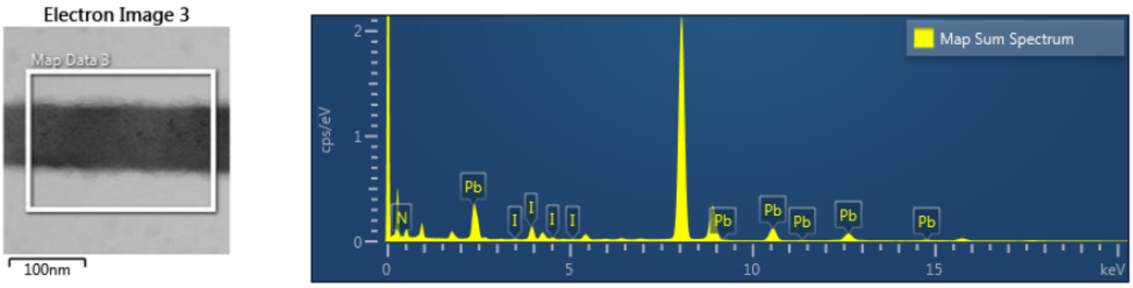

Element	Line Type	k factor	Absorption Correction	Wt%	Wt% Sigma	Atomic %
N	K series	3.69981	1.00	2.41	1.08	20.47
I	L series	2.00294	1.00	64.47	1.45	60.49
Pb	L series	1.67913	1.00	33.13	1.31	19.04
Total:				100.00		100.00

Supplementary Figure 8. EDX spectra of a 100-nm-wide perovskite $CH_3NH_3PbI_3$ nanowire. The 100-nm-wide single-crystal perovskite is used to investigate the stoichiometric ratio in the entire region. The atomic ratio of N:Pb:I at the table is approximately 1:1:3, which is the theoretical stoichiometric ratio of the $CH_3NH_3PbI_3$. The Cu peaks at 8 keV originate from the TEM grid. This stoichiometric data suggests that the perovskite single crystals have the compositional structure.

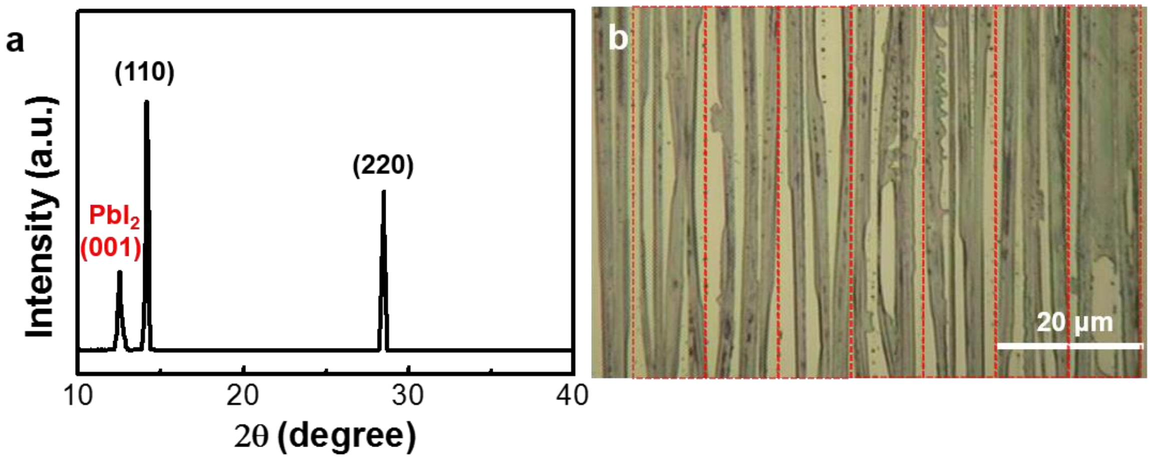

Supplementary Figure 9. The temperature effect on the properties of perovskite patterned thin films. (**a**) The XRD scan of the perovskite patterned thin film prepared at 220 °C. At this high temperature, a lead iodide peak appears due to thermal degradation of the perovskite. (**b**) The OM image of the perovskite patterned thin film prepared at 150 °C. The narrow needle shape crystals are observed in the 10-μm-wide channel because probably more nuclei are formed during the slow crystal propagation. The red dot lines indicate a channel width of 10 μm.

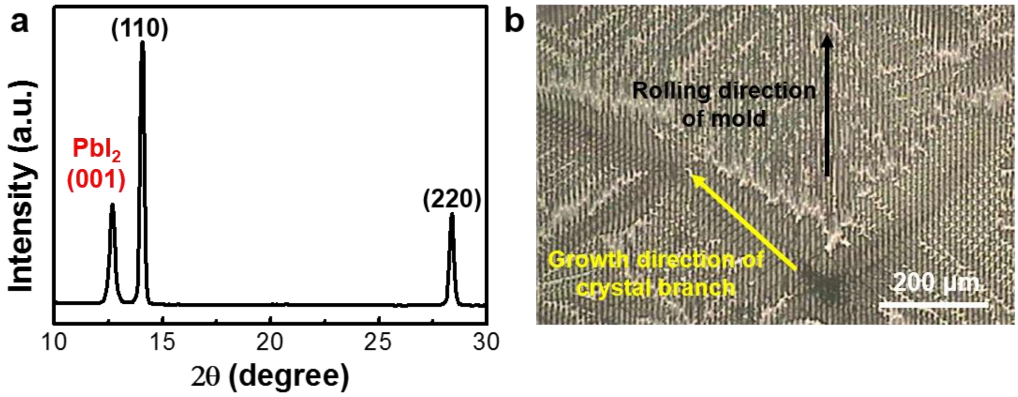

Supplementary Figure 10. The influence of rolling speed on the single-crystal perovskite thin films produced via GC-LCG. (**a**) XRD profile of perovskite patterned thin film produced with a rolling speed of 200 μm s^{-1}. Compared to the optimized rolling speed (1 mm s^{-1}), it takes longer to complete the process with this slower rolling speed. The PbI_2 (001) diffraction peak indicates the degradation of $CH_3NH_3PbI_3$ (converted to PbI_2) caused by the longer thermal exposure, which suggests the failure of the GC-LCG process at a rolling speed of 200 μm s^{-1}. (**b**) OM image of perovskite patterned thin film with branched patterns produced at a rolling speed of 3 mm s^{-1}. Due to the incomplete crystallization caused by the rapid removal of the channels, growth of the crystals is not restricted along the channel but propagates as a branched shape; this suggests the failure of the GC-LCG process at a rolling speed of 3 mm s^{-1}.

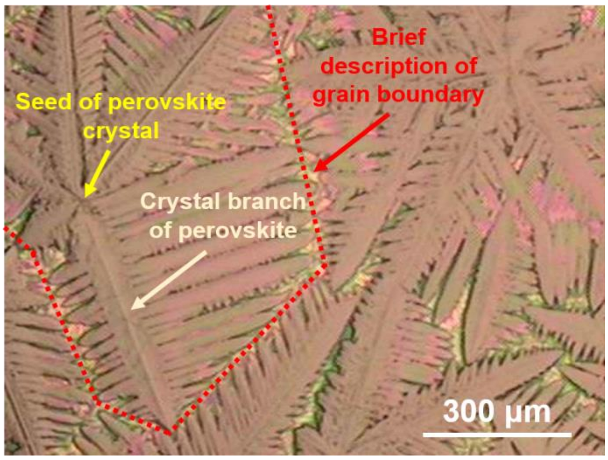

Supplementary Figure 11. OM image of a polycrystalline perovskite thin film prepared using a flat PDMS sheet. The perovskite crystals grow in all directions from the seed of the perovskite crystal to form branched shapes due to the absence of geometrical confinement. The branched crystal size is on the scale of a few hundred microns, as marked by the dotted red line.

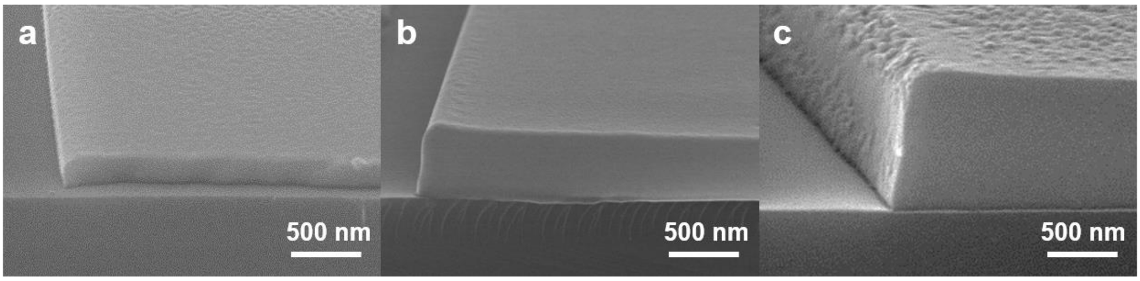

Supplementary Figure 12. The vertical confinement effect of the perovskite crystals fabricated from molds with various channel depths. (**a**), (**b**), (**c**), Cross-sectional SEM images of (**a**) 200-nm-thick, (**b**) 500-nm-thick, and (**c**) 1-µm-thick $CH_3NH_3PbI_3$ perovskite crystals. In comparison to the smooth morphology of the 200- and 500-nm-wide crystals, agglomerates are observed on the surface for the 1-µm-wide perovskite crystal. The channel widths of all samples are 10 µm.

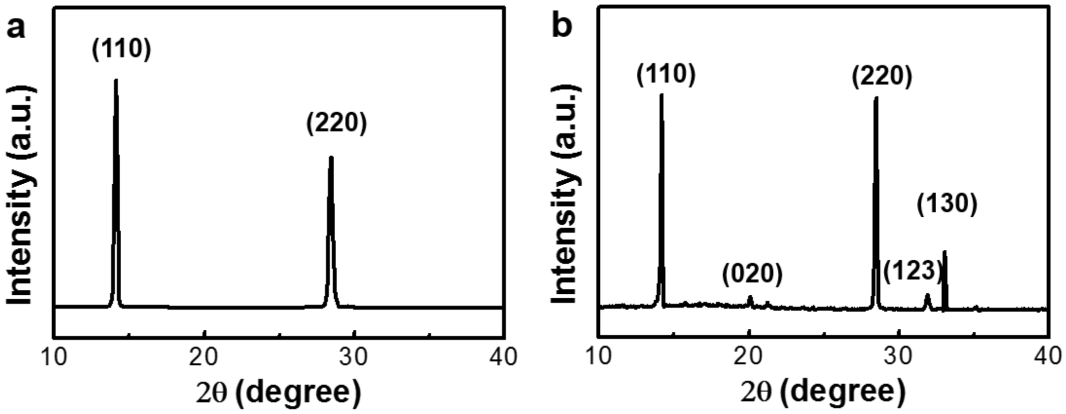

Supplementary Figure 13. Out-of-plane XRD analysis of perovskite thin films with different thicknesses. (**a**) XRD profile of the 500-nm-thick $CH_3NH_3PbI_3$ perovskite patterned thin film. The strong diffraction peaks at the (110) and (220) lattice planes indicate crystal growth along the [110] axis, which is caused by the successful GC-LCG process. (**b**) XRD profile of the 1-μm-thick perovskite patterned thin film. The small diffraction peaks of the (020), (123), and (130) planes indicate the existence of a tilted growth facet along the surface normal direction, which suggests failure of the vertical confinement.

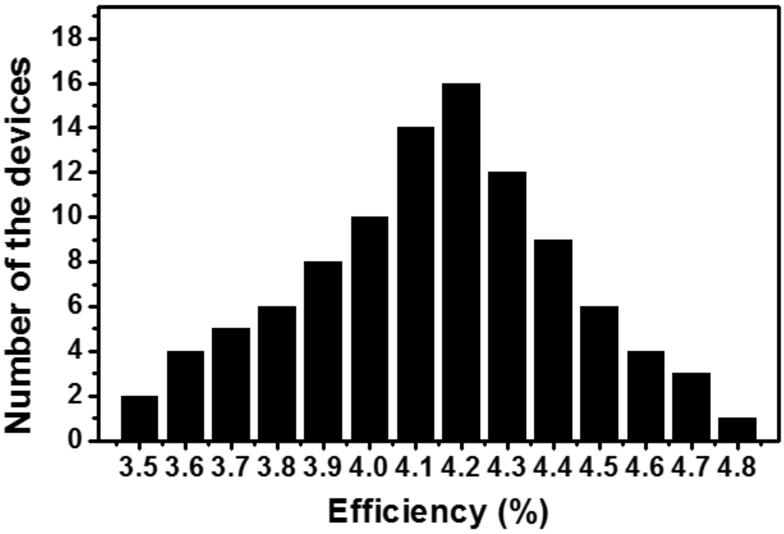

Supplementary Figure 14. Histogram of active-area PCEs obtained from 100 perovskite lateral perovskite solar cells. The average efficiency is 4.14%, and the maximum efficiency is 4.83%. The low distribution of the device efficiency (ca. ± 0.7%) indicates the high reproducibility of the devices made with the GC-LCG-processed single-crystal perovskite thin films.

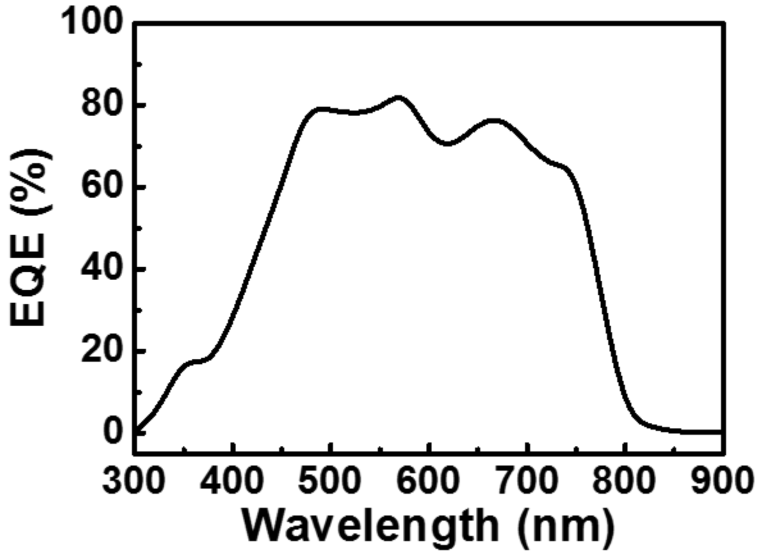

Supplementary Figure 15. The EQE spectrum of a single crystalline perovskite thin film lateral perovskite solar cell under the light spectrum of AM 1.5G. The EQE is up to 80% in the broad spectral range of 450 nm to 750 nm. Before the EQE measurement, a poling process was carried out to form the p-i-n structure by inducing ion migration in the perovskite. The poling voltage was fixed at 0.39 V μm^{-1} which is identical to the poling voltage for the PCE measurements. After the poling, the EQE spectrum of the solar cells was measured under short circuit condition with bias light.

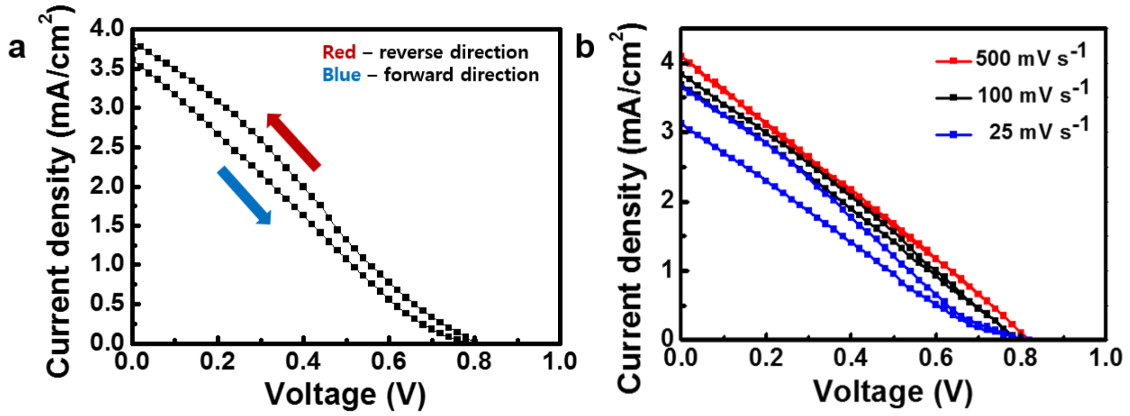

Supplementary Figure 16. *J*-*V* curves of the single crystalline perovskite thin film lateral perovskite solar cells. All of the experiments are measured under light intensity of 12.2 mW cm^{-2}. (**a**) *J*-*V* curve of the single crystalline perovskite thin film lateral perovskite solar cell. The measurement was performed from reverse direction (red arrow) to forward direction (blue arrow). Active-area PCE from the measurement through reverse and forward direction is 6.60% and 5.43% with a J_{sc} of 3.84 mA cm^{-2} and 3.61 mA cm^{-2}, a V_{oc} of 0.802V and 0.782 V, respectively. (**b**) *J*-*V* curves of the single-crystal perovskite thin film obtained with different scan rates of 25, 100, and 500 mV s^{-1}. The hysteresis is observed under the slow scan rate of 25 mV s^{-1}, which may be caused by the polarization at the interface of electrode/perovskite [5].

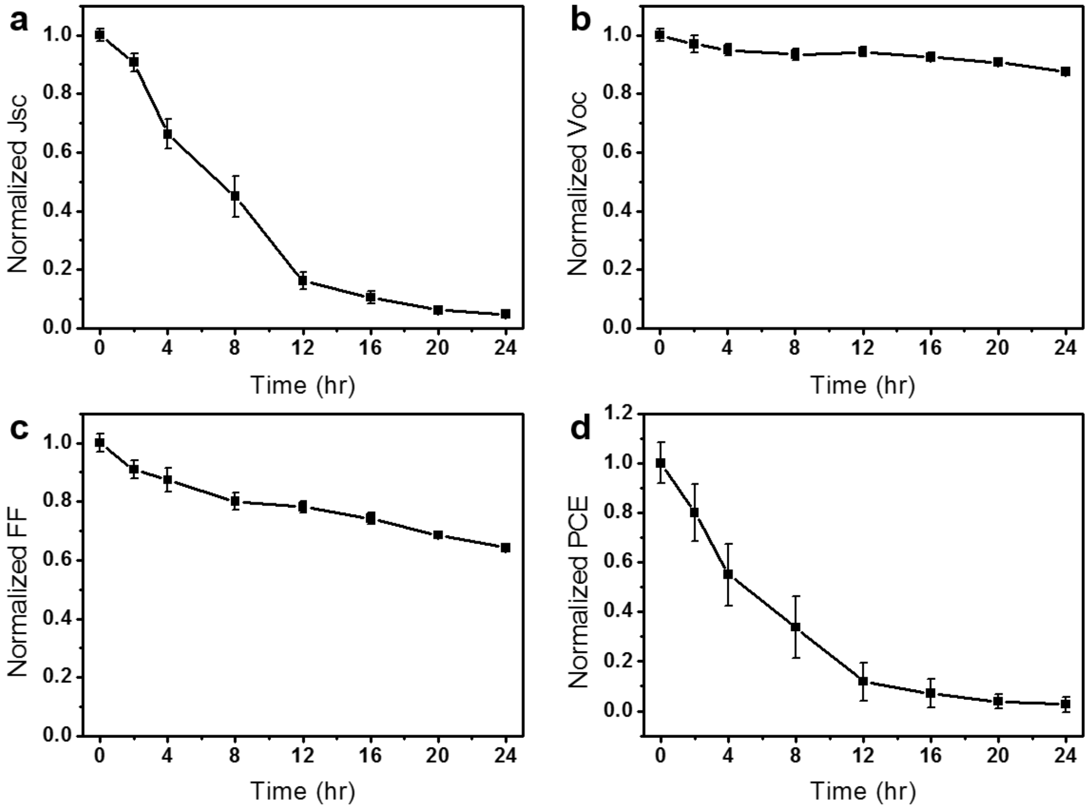

Supplementary Figure 17. The overtime evolution of ten perovskite lateral perovskite solar cell devices. All of the experiments are measured under light intensity of 12.2 mW cm^{-2}. Normalized (**a**) J_{sc}, (**b**) V_{oc}, (**c**) fill factor, and (**d**) Active-area PCE as a function of time. The devices were measured and stored in an ambient environment (25 °C temperature and 30-50 % humidity).

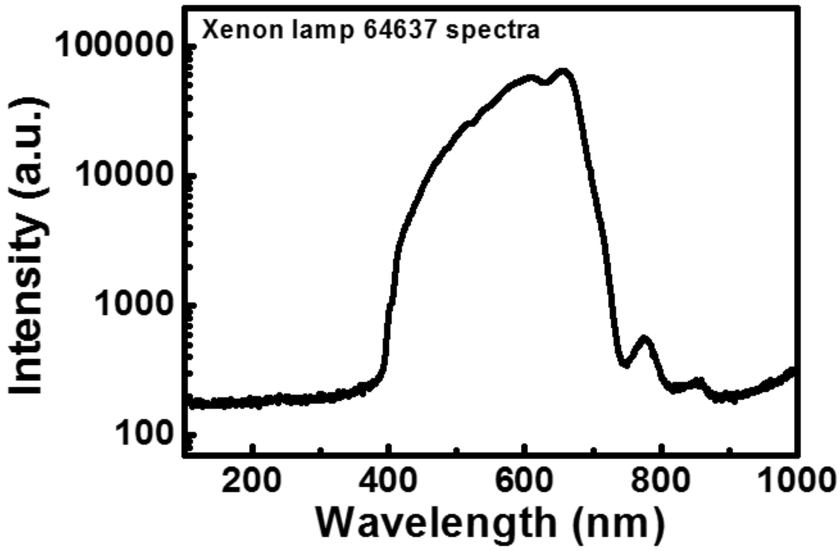

Supplementary Figure 18. The light source spectra profile of Xenon lamp 64637 for the experiments using low light intensity (12.2 mW cm^{-2}). The spectrum was measured using Si photodiode. The average light intensity of the lamp is 12.2 mW cm^{-2}.

Supplementary References

1. Ha, S.T. et al. Synthesis of organic–inorganic lead halide perovskite nanoplatelets: towards high-performance perovskite solar cells and optoelectronic devices. *Adv. Opt. Mater.* **2**, 838-844 (2014).
2. Kim, M. et al. Lateral organic solar cells with self-assembled semiconductor nanowires. *Adv. Energy Mater.* **5**, 1-8 (2015).
3. Nie, W. et al. High-efficiency solution-processed perovskite solar cells with millimeter-scale grains. *Science* **347**, 522-525 (2015).
4. Shao, Y., Xiao, Z., Bi, C., Yuan, Y. & Huang, J. Origin and elimination of photocurrent hysteresis by fullerene passivation in CH3NH3PbI3 planar heterojunction solar cells. *Nat. Commun.* **5**, 1-7 (2014).
5. Chen, B., Yang, M., Priya, S. & Zhu, K. Origin of J–V hysteresis in perovskite solar cells. *J. Phys. Chem. Lett.* **7**, 905-917 (2016).
